# Characterization of Phosphate Solubilizing Bacteria in Sediments from a Shallow Eutrophic Lake and a Wetland: Isolation, Molecular Identification and Phosphorus Release Ability Determination

**DOI:** 10.3390/molecules15118518

**Published:** 2010-11-22

**Authors:** Yichao Qian, Jiyan Shi, Yingxu Chen, Liping Lou, Xinyi Cui, Rukun Cao, Pengfei Li, Jie Tang

**Affiliations:** Key Laboratory for Water Pollution Control and Environmental Safety, Institute of Environmental Science and Technology, Zhejiang University, Hangzhou 310029, Zhejiang Province, China

**Keywords:** sediment, phosphorus, phosphate solubilizing bacteria, 16S rDNA, *Cupriavidus basilensis*

## Abstract

The transformation of phosphorus (P) is a major factor of lake eutrophication, and phosphate releasing bacteria play an important role in the release process. Experiments were conducted to investigate P content and characterize phosphate solubilizing bacterial composition at the molecular level in a shallow eutrophic lake and a wetland. Results showed that P concentrations were relatively high and derived from agricultural runoff and domestic or industrial pollution. Enumeration and molecular identification of these strains indicated that these bacterial groups were abundant in the ecosystem and various kinds of bacteria participated in the phosphorus release process. Twelve phosphate solubilizing bacteria, including eight organic P-solubilizing bacteria (OPBs) and four inorganic P-solubilizing bacteria (IPBs), which belonged to three different families, were isolated and identified. *Cupriavidus basilensis* was found for the first time to have the ability to mineralize organic P (OP). Laboratory tests on P release ability revealed that IPBs were more effective at releasing P than OPBs. The most efficient IPB strain could accumulate over 170 mg·L^-1^ orthophosphate, while the equivalent OPB strain only liberated less than 4 mg·L^-1^ orthophosphate in liquid culture. The results obtained from this investigation should help clarify the roles of microorganisms in aquatic systems and the mechanisms of eutrophication.

## 1. Introduction

Phosphorus (P) is proven to be one of the key limiting factors of bacterial activity and for primary production in aquatic systems [1]. With the constant input of sewage and fertilizer in past years, large amounts of P have been detected accumulating in lake sediments and would be released into overlying water under suitable environmental conditions, a process known as internal P loading [2]. The release of accumulated P may contribute a substantial amount compared to external sources and can maintain lakes in a eutrophic state for extended periods of time, even when the external loading is reduced [3]. It is therefore of great interest to clarify the process and mechanism(s) of P release.

Not all forms of P tend to become released from sediment, and therefore the bioavailability of P is closely related to the various forms of P present [4]. Accordingly, detailed knowledge of the different chemical P fractions in lake sediments is essential for understanding the release process at the water-sediment interface. Many sequential chemical extraction procedures have been applied to estimate the mobility and bioavailability of P in soils and sediments [5,6]. However, there is no standard method. Researchers always choose specific procedures according to different requirements. For the P composition in sediments of shallow lakes, the Standards Measurements and Testing Program of the European Commission (SMT protocol) is widely used and the graded P fractions are also useful to analyze the potential release and possible sources of P in sediments [7].

Although the transition and transformation of P at the water-sediment interface has received much attention, knowledge of this field is still insufficient [8]. The release process and mechanism(s) are of physical, chemical, and biological nature. A substantial amount of research has been carried out in the chemical and physical fields, such as the impact of changes in pH [9], dissolved oxygen [10], and disturbances [11]. In the biological field, there are many studies supporting the view that microbial activity can significantly influence the release of P [12,13]. Indeed, bacteria can dissolve insoluble inorganic P (IP) with the action of low molecular weight organic acids which are produced in the periplasmic space of some Gram-negative bacteria through a direct glucose oxidation pathway [14]. Digenesis of organic matter is mainly dependent on bacterial activity since the mineralization of these compounds is carried out by means of several phosphatases which are secreted by microorganisms [15]. These groups of bacteria that can directly influence P release have been termed ‘phosphate solubilizing bacteria’. Previous studies with respect to the relationship between P and phosphate solubilizing bacteria have mainly focused on these microorganisms’ role in plant growth promotion and enhanced biological phosphate removal [16,17]. For lake environments, different kinds of phosphate solubilizing bacteria have been isolated and identified, and tests of P releasing ability has proven that these bacteria indeed playan important role in the P release process [18]. Furthermore, other sediment studies have also paid attention to the variations of alkaline phosphatase activity, which is highly related to the bacterial metabolism and orthophosphate concentrations in eutrophic lakes [19]. However, compared to the study depth of phosphate solubilizing bacteria and the abundance of their library in soil, the identified species in sediments are quite inadequate and the detailed information about bacteria, especially concerning the bacterial diversity at different sediment depths and specific microorganisms capable of dissolving inorganic and organic P, is often lacking.

Therefore, in this study we took sediments from a shallow lake and a wetland as representative aquatic environments. Different P fractions were analyzed using the SMT protocol, and the vertical abundance of phosphate solubilizing bacteria was studied for different sampling sites. Moreover, the specific bacteria were isolated by a culture-dependent method and identified by their 16S rDNA sequences. The selected bacterial release abilities of IP and OP were also tested. The objective of this work was to enrich our knowledge of the phosphate solubilizing bacterial library in sediments and make their roles in P transfer and the mechanism of lake eutrophication more clear.

## 2. Results

### 2.1. Vertical distribution of P in sediment

**Figure 1:**
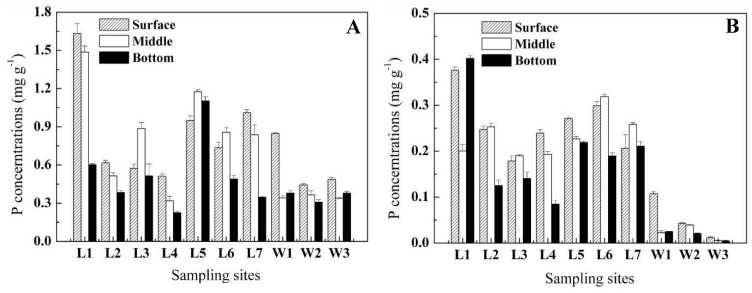

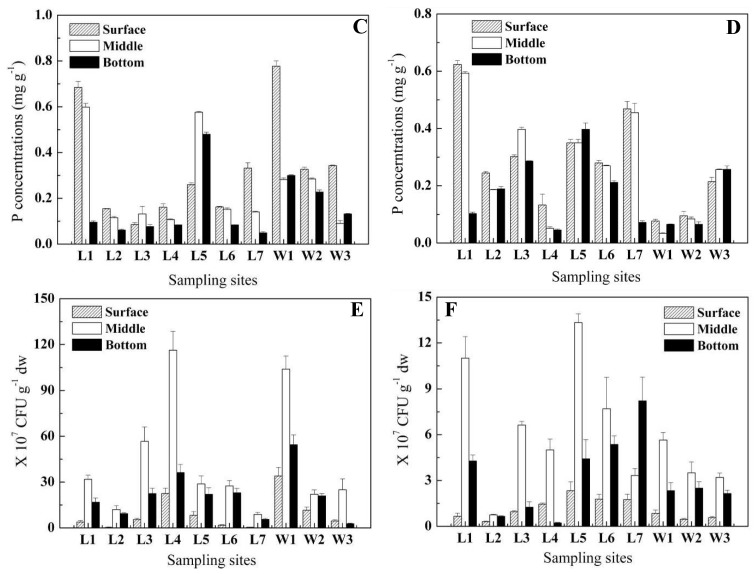
Vertical variations of concentrations of different P fractions and distribution of phosphate solubilizing bacteria in different sampling sites (A: TP; B: OP; C: NaOH-P; D: HCl-P; E: OPB B; F: IPB).

Results of the vertical variations of P and bacterial contents in sediment are illustrated in Figure 1. On average, the concentration of TP was 0.75 ± 0.38 mg g^-1^ in lake sediment, and 0.43 ± 0.16 mg g^-1^ in wetland sediment (Figure 1A). OP was the primary factor resonsible for the TP content differences between the lake and wetland sediments (Figure 1B). Only about 6.4% (1.2%-12.6%) of TP existed combined with organic matter in the wetland sample, while an average of 35.5% (19.8%-66.8%) was found combined with organic substances in lake sediment. IP, which is represented as NaOH-P and HCl-P, was also an important form of P in both lake and wetland samples, although the dominant form was different in each case: NaOH-P made up a relative high percent of wetland IP, especially in surface sediments (Figure 1C), while HCl-P, mainly found in surface and middle sediments, accounted for over 60% of IP in the lake samples (Figure 1D). 

### 2.2. Abundance of IPB and OPB in sediment

After 3-day and 7-day growth of OPBs and IPBs in relevant agar, the total number of bacteria was counted, and the microbial plate count results are given in Figures 1E and F. The illustration shows that bacterial numbers varied greatly among different samples.

OPB numbers ranged from 0.35 ± 0.21×10^7^ to 116.25 ± 12.37 ×10^7^ CFU g^-1^ dry sediment in lake, and from 2.75 ± 0.35×10^7^ to 104.00 ± 8.48×10^7^ CFU g^-1^ dry sediment in wetland. The counts of IPBs grown on calcium phosphate agar ranged from 0.23 ± 0.04 ×10^7^ to 8.22 ± 1.55 ×10^7^ CFU g^-1^ dry sediment in lake, and from 0.46 ± 0.06 ×10^7^ to 5.65 ± 0.49×10^7^ CFU g^-1^ dry sediment in wetland. In general, the OPB numbers were one order of magnitude higher than in IPBs. However, the vertical distribution characteristics of these two bacterial species were almost same in that few bacteria accumulated on the surface and plentiful phosphate solubilizing bacteria existed in the middle parts. In addition, there was no remarkable correlation between P content and the bacteria numbers in the same samples. 

### 2.3. Identification of P releasing bacteria

**Figure 2:**
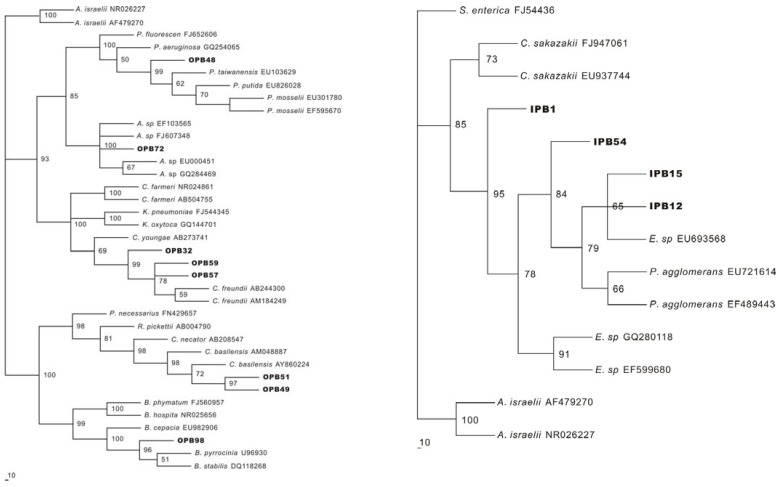
Phylogenetic analysis of OPB and IPB based on partial 16S rDNA sequence and those stored in public nucleotide databases. The tree was constructed by using Paup v.4.0b.8.a Software. Distance matrix was calculated by Kimuras two-parameter model. Bootstrap values based on 1,000 replications are listed as percentages at the branching points.

Among over 100 isolates, 12 predominant strains comprising eight OPBs and four IPBs performed well in plate assays and exhibited significant P solubilization in liquid cultures. The identification of these bacteria was based on partial 16S rDNA gene sequences, and their phylogenies are presented in Table 1 and Figure 2. The 16S rDNA sequence analysis placed all four of the IPB isolates within *Enterobacter sp.* and *Pantoea agglomerans,* which were in one family, *Enterobacteriaceae*. Isolations of bacteria belonging to this family have already been detected in various soils and discovered to have IP solubilizing abilities [20]. 

**Figure 3:**
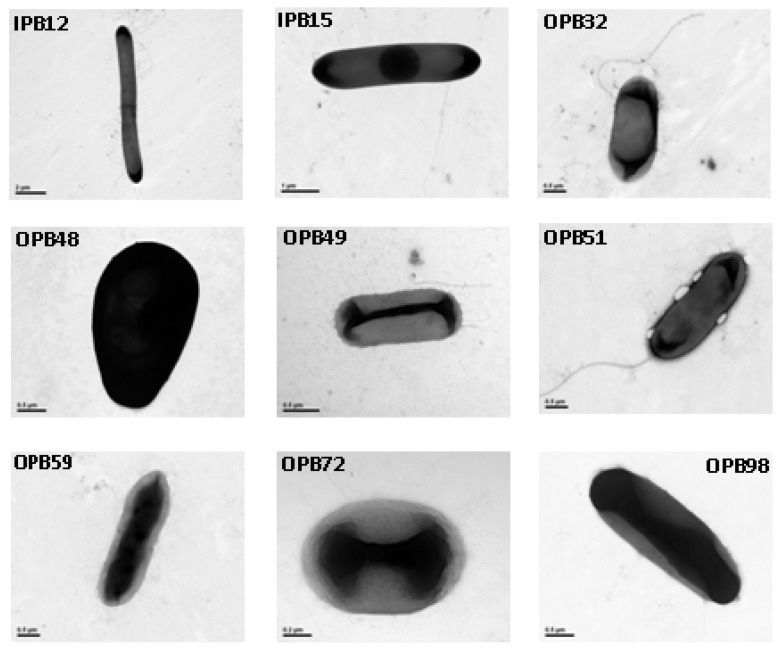
The morphological characterization of OPBs and IPBs under the transmission electron microscope (TEM).

OPB 32, OPB 57, and OPB59 were highly related to *Citrobacter freundii*, which is a straight bacilliform bacterium with a flagellum to maintain mobility (Figure 3) and that uses citrate as its only carbon source. 

OPB49 and OPB51 displayed relatively low similarities with their closest matches (96% and 98%, respectively), but their morphological and biochemical characteristics matched well with *Cupriavidus basilensis* (Figure 3 and Table 2), a bacterium belonging to the genus *Wautersia* reportedly able to resist metals and decompose some dissimilation materials which were difficult to degrade [21]. This strain, being reported here for the first time as a phosphate solubilizing bacteria, possesses the ability to mineralize considerable amounts of organic P. OPB48 and OPB98, closely matched *Pseudomonas sp.* and *Burkholderia sp.*, which are regarded as common phosphate solubilizing bacteria. OPB72 was closely related to *Acinetobacter sp.*, a strictly aerobic nonfermentative Gram-negative bacillus with significant contributions in the mineralization of, for example, aromatic compounds [22].

### 2.4. P release ability of predominant phosphate solubilizing bacteria

**Figure 4:**
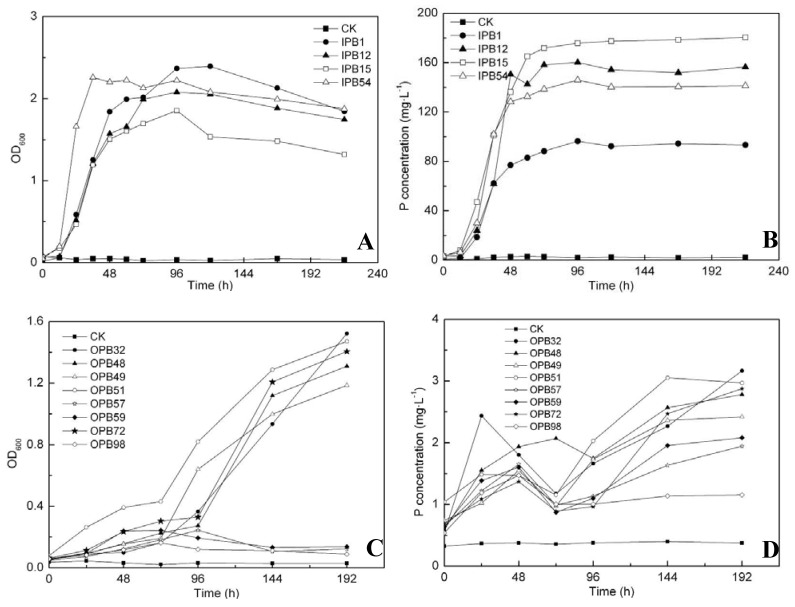
The calcium phosphate solubilizing abilities of IPBs and lecithin mineralizing abilities of OPBs. Bacterial growth is evaluated by OD_600_ of the culture, which also represent the phosphate assimilated by microbes, and P concentrations represent the orthophosphate dissolved in the liquid.

The IP/OP release abilities of different bacteria strains are shown in Figure 4. Optical density of the culture liquid was determined at 600 nm (OD_600_) to estimate bacterial growth yield and P concentration was represented as the contents of orthophosphate in liquid medium. P release ability could be evaluated from liberated P, which either dissolved in supernatant in the form of orthophosphate or was assimilated by microorganisms to form cell tissues. 

Because of their homology, the four IPBs exhibited similar growth patterns. All of them entered a rapid multiplication phase immediately after the experiments started, moved into the stationary phase after 48 h, and began to decline on the fourth day (Figure 4A). These growth curves agreed perfectly with the typical curve which moved from log phase to stationary phase, then to death phase, while the lag phase was very short. The variations in P concentrations were quite similar to the growth situation (Figure 4B). The content of orthophosphate increased significantly in the first two days with the rapid growth of bacteria and then remained almost steady for the remainder of the experiments. Moreover, strain IPB1 grew the fastest among four IPBs, but it accumulated the least P in culture, while IPB15 behaved just the opposite. This suggested that most of the released P was utilized by IPB1 to form part of the cell constituents, while for IPB 15, the assimilation was relatively weaker. As for OPBs, they both had a longer lag phase (Figure 4C). Strain OPB 32 (OPB 57, OPB 59) and OPB 48 started to bloom after 96 h, and the beginning of OPB 49 and 51 was at the third day, whereas OPB 98 grew very slowly and accumulated a small amount of bacterial biomass. All the P concentration curves presented a trend of rapid increase during the first 24 hours, followed by a decrease to the original level at the end of the third day, and then increased again to different levels according to the individual characteristics. In general, OPBs grew slower and liberated less orthophosphate than IPBs. By comparing the P release abilities of the most efficient bacteria, OPB 32 and IPB 15, it was concluded that the IPB strain could accumulate over 170 mg L^-1^ orthophosphate, while the OPB strain could accumulate less than 4 mg L^-1^ orthophosphate in liquid culture, and the biomass of OPB in lecithin media was also lower than that of IPB.

## 3. Discussion

According to the SMT protocol, P in the West Lake and the Xixi Wetland was mainly of anthropogenic origin, since NaOH-P plus OP accounted for around 60% and 80% of TP, respectively [23]. Moreover, among the possible sources of these allochthonous P, lake P may come from agricultural runoff (NaOH-P + OP), while wetland P maybe from domestic or industrial effluents (mostly NaOH-P) [24]. HCl-P was another important P existing form, which reached an average of 36.7% of TP in lake sediment. Therefore, mineralization of OP, solubilization of HCl-P and separation of NaOH-P would be important release pathways of internal P loadings in these two shallow waters. 

The mechanisms involved in the transformation of different P species are of biological, chemical and physical nature. In the biological area, bacteria were proven to play an important role in P solubilization and mineralization processes [25]. In this study, the pH in the supernatant of the IPB incubations was observed to decrease during the release experiments (while being almost stable in the control incubation), which was of benefit to scavenge P from insoluble mineral sources. Interestingly, along with the drop of pH in the last period of the experiments, the growth of IPBs stopped; even some dead IPBs began to appear (Figure 4A). This suggested that an acidulous/acidic condition might be an unsuitable environment for IPBs’ growth. Hence it could be extrapolated that production of organic acid lead to acidification of microbial cells and their surroundings, and then orthophosphate was released from mineral phosphate by proton substitution for Ca^2+^, whereas when pH dropped to a certain degree, the process of solubilization would be restricted (but the threshold for this has not been determined). In the West Lake, the pH of the water bodies was slightly alkaline, which was suitable for IPBs’ growth but not suitable for the liberation of HCl-P. Moreover, frequent disturbance in lake water would weaken the acidification induced by IPBs at the water-sediment interface. It was therefore not surprisingly to find a great quantity of IPBs and relatively high content of HCl-P in lake sediment (Figure 1). As for the Xixi Wetland, abundant IPBs but deficient HCl-P under alkalescent conditions indicated that the acidification might not be the only factor for P liberation. Chelation of a phosphate cation by organic metabolites was presumed to be another important release route. Studies have shown that although bacteria grew weakly in alkaline conditions, they also could liberate P by producing organic metabolites in special soils with pH=12 [26]. 

**Figure 5:**
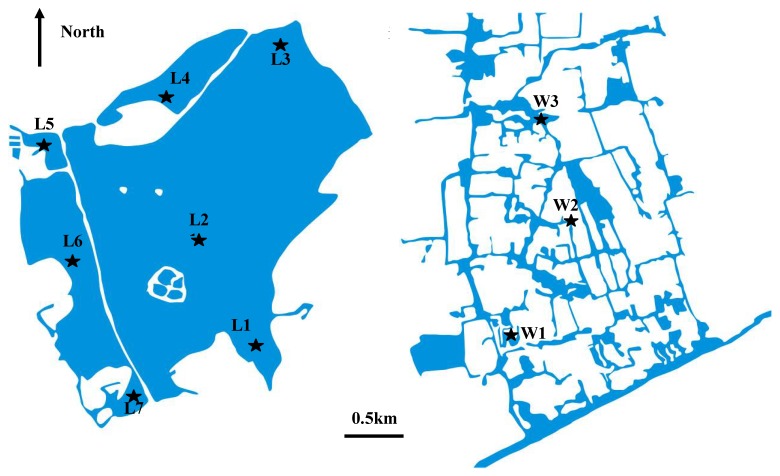
The geographic location of the sampling sites.

The sediment contained a large number of organic substances, which were the source of P for hydrophytes. However, most of OP could not be assimilated directly by phytoplankton but could be transformed to an available form by different types of enzymes [27]. In fact, the primary source of these enzymes was considered to be of microbial origin and the synthesis was regarded as two modes, inducible and constitutive. Nonspecific alkaline phosphatases, enzymes that hydrolyzed phosphate esters [28], were one group of inducible enzymes and their synthesis and activities were highly controlled by the ambient P nutrition. In this study, we used lecithin as the sole OP source in liquid medium and found that the P concentration curves presented an “N” shape (Figure 4D). This was mainly due of the interactions of orthophosphate and phosphatases. In the early stage of the experiments, the activities of alkaline phosphatases were strongly stimulated due to the low contents of orthophosphate, but they were gradually inhibited accompanied by an increase of orthophosphate concentration in supernatant. When the mineralization was too weak to balance the assimilation and catabolism of bacteria, orthophosphate content began to decrease while phosphatase activity started to recover, and then the relation of ebb and flow continued. In the West Lake and the Xixi Wetland, large amounts of bacteria were detected to be involved in OP transformation (Figure 1), whereas laboratory tests confirmed that the selected OPB strains only maintained less than 4 mg·L^-1^ orthophosphate under suitable conditions (Figures 5C and D), illuminating further that the most of the enzymes participating in the process of mineralization were inducible components. On the other hand, nonspecific acid phosphohydrolases, including 3’-nucleotidases and 5’-nucleotidases [29], were thought to be constitutive and P irrepressible. These phosphatases were produced mainly to serve the internal P metabolism and seemed to play less important role in external OP decomposition in alkaline environment. 

It has been reported that the population of phosphate solubilizing bacteria was considerable in sediment and soil, including both aerobic and anaerobic strains, such as *Pseudomonas*, *Bacillus*, *Rhizobium*, *Burkholderia*, *Achromobacter*, *Agrobacterium*, *Micrococcus*, *etc*., and new strains are constantly being discovered. For example, *Cupriavidus basilens* was proven to have the ability to decompose OP in the present study. PCR based techniques and fluorescence *in situ* hybridization (FISH) were proven to be powerful tools for charactering the constituents of sediment communities. However, because of a great variety of phosphate solubilizing bacteria, it was very difficult to find a section of conserved gene sequences in every single bacterium in order to design suitable probes for molecular analysis. In addition, an understanding of the properties, regulation, and role of the phosphatase enzymes, which were directly involved in P mineralization and solubilization, was still hazy [30]. Therefore, in order to explore the functions and quantify the release abilities of phosphate solubilizing bacteria, the plate screening methods were still necessary. In this study, we isolated 12 phosphate solubilizing bacteria, including eight OPBs and four IPBs. Phylogenetic analyses illustrated that the bacteria from various families participated in P cycle in these shallow waters. Among the eight OPB strains, OPB 32, OPB57 and OPB 59 belonged to the same species *Citrobacter freundii*, of the family *Enterobacteriaceae*. 

Reports have already revealed that bacteria belonging to this family were capable of effectively producing different patterns of phosphatases and then mineralized macromolecular organic P into orthophosphate [31]. OPB 48 and OPB 72 were in the family *Pseudomonadaceae,* which could produce alkaline phosphatase under conditions of low P availability, but the activity would be repressed by a high content of orthophosphoate [32]. From the analysis results of phylogenetic, OPB 49 and OPB 51 closely matched *Cupriavidus basilensis*, which was once named as *Wautersia* and to our knowledge, no previous research has found this strain to possess of the ability of OP mineralization. For quality assurance, the competitive studies on biochemical characteristics of OPB 49, OPB51 and *Cupriavidus basilensis* have performed and the results proved further that they had very high similarity (Table 2). *Cupriavidus basilensis* is a Gram-negative flagellated bacterium capable of copper chelation, but the mechanisms of OP decomposition have not yet been clarified. OPB 98 highly matched *Burkholderia cepecia*, a high G + C percentage Gram-negative bacterium which was reported to be capable of degrading calcium phosphate [33], but in this study, it also showed the capability of degrading OP. Previous studies have found out that P solubilization and mineralization could coexist in the same bacterial strain, whereas the mechanisms were not clear [34]. Genetic mechanism is the probable reason, since the production of phosphatase and organic acid was assumed to be controlled by the expression of some certain genes, and such expression could be regulated by different substrates [31]. Therefore, the types of gene expression might be determined by the available substrates, thereby affecting the production and activity of phosphatases and organic acid. As for IPBs, all the IPBs were in the genus *Enterobacter,* a common bacterium involved in solubilization of IP [35]. The mechanisms of inorganic P solubilization have been discussed above. The existence of mineral P solubilization genes was demonstrated and some of them have already been isolated from several bacterial species. However, the specific genes that involved in organic acid synthesis in *Enterobacter* have not been reported. In conclusion, the available evidence indicates that the genetic manipulation is the fundamental factor that controls the procedure of P decomposition, thus considerable future researches are needed to be developed to identify more relevant genes and elucidate the genetic mechanisms in P bio-transformation.

## 4. Experimental

### 4.1. Description of the study area

West Lake, located at 120°16´ east and 30°15´ north, is a typical urban shallow lake with an average depth of 2.2 m. The watershed area of the lake is about 21 km^2^, and the water surface area is 6.5 km^2^. The lake comprises five sub-areas, named Outer, Beili, Yue, Xili, and Xiaonan (Figure 5), separated by three causeways. Nine bridges were built on the causeways to allow the interchange of water. In recent decades, with the increase of population and development of the economy in its catchment area the lake has undergone serious eutrophication. Although a dredging project was completed in 2003, the water quality remains deteriorated. According to an investigation in 2003, total nitrogen (TN) and total P (TP) in the water were 2.21 mg·L^-1^ and 0.12 mg L^-1^, respectively, with the TP concentration exceeding considerably the U.S. Environmental Protection Agency (USEPA) 0.025 mg L^-1^ upper limit of standard lake eutrophication Xixi Wetland, known as the “kidney” of the city of Hangzhou, is located in the west of Hangzhou city and is less than 5 km away from the West Lake. It is a rare urban secondary wetland and the only national wetland park which has integrated urban, agricultural and cultural wetlands. The area of the wetlands is about 10 km^2^, and 70% of the area is composed of rivers, ponds, marshes and other water bodies. As a result of pollution from agriculture and urban life, nitrogen and P are the main pollutants in water, and in some places, the concentrations of TP are over 0.25 mg L^-1^ [36]. However, detailed studies on its sediments have not yet been conducted.

### 4.2. Sediment sampling

Representative sites in the West Lake and the Xixi Wetland were selected for this study. Sites L1-L9 are in the lake, and sites W1-W3 are in the wetland (Figure 5). Site L3 and L7 are located at the outlet and inlet of the lake, with water flowing from southwest to northeast. Site L1 and L6 are located at the entrance of the streams, where sediments are mostly polluted by nutrient elements. Site L4 and L5 are situated at corners in the direction of the water flow, where the water quality is lower because of poor water mobility. Site L2 is the center of the lake. The water depth is very low at the site W1; thus redox conditions of the surface sediment could change frequently. Site W2 and W3 are located at the regions with better water quality, and large emerging plants, such as reeds, dominate the latter site.

At each site, three sediment cores were taken with a gravity core sampler (Nanjing Institute of Geography and Limnology, Chinese Academy of Sciences, NIGLAS) and a home-made sampling tube. The tube was 5 cm in diameter and 40 cm long with sealed holes at certain positions along the wall. Sediment samples could be collected from the holes very quickly in order to prevent changes in elemental speciation due to the influence of different environmental conditions. All the sediment samples were sealed and kept on ice, then carried back to the laboratory within two hours. In the laboratory, sediment samples were collected from the holes at depths of 0, 20 and 40 cm, which represented the surface, middle and bottom of the sediment column core. As the deposition rate of the West Lake was about 0.39 cm a^-1^, such sampling methods could exhibit the situations in 50 and 100 years. Samples for sedimentary P analysis were then freeze-dried (Christ Alphal-4LSC, Germany) and put through a 149 µm filter prior to chemical analysis. Samples for phosphate solubilizing bacterial enumeration were kept at 28°C and made into aqueous extracts within two hours.

### 4.3. Analysis of P fraction

Determination of P fractions was done using an analytical protocol developed by the SMT protocol. A sediment reference material (SRM, GBW07301a, 1520 ± 70 µg g^-1^ sediment, certified by the China Standards Bureau) was used to confirm the accuracy of the SMT procedure. All samples were analyzed in triplicate, and the data were expressed as means and standard deviations.

### 4.4. Enumeration of phosphate solubilizing bacteria

To enumerate IPBs and OPBs, aqueous extracts of fresh sediment samples (5 g) were serially diluted and smeared onto IP agar (glucose, 10.0 g; (NH_4_)_2_SO_4_, 0.5 g; NaCl, 0.3 g; KCl, 0.3 g; FeSO_4_, 0.03 g; MnSO_4_, 0.03 g; MgSO_4_, 0.3 g; pH 7.2; distilled water, 1,000 mL) with calcium phosphate [Ca_3_(PO_4_)_2_, 1.0 g] as sole P source. OP agar was similar to IP medium except that yolk including lecithin (40 mL) was added instead of calcium phosphate. Plates of IPBs and OPBs were incubated for 3-7 days (28 °C, dark, oxic) prior to counting the numbers of colony forming units (CFU), and colonies with unique morphology were picked up and purified.

### 4.5. Determination of P release ability

P release ability was quantitatively estimated by inoculating the isolated bacterium to IP (same as above, without agar) and OP liquid medium (similar to above, with lecithin added instead of yolk and without agar). After culturing at 28°C for predefined time, the cultures of IPBs were shaken emphatically to set free the adherent bacteria and then left to stand still for 10-15 min in order to precipitate calcium phosphate. After that, optical densities of the culture liquid were determined at 600 nm (OD_600_) with a Biophotometer (Eppendorf, Germany) to evaluate IPBs’ and OPBs’ growth conditions, and then centrifuged at 12,000 rpm for 10 min to remove bacterial biomass. Orthophosphate content in the supernatant was determined using the molybdate blue method. P release ability was expressed according to the bacterial growth yield and concentration of orthophosphate accumulated in the culture solution.

### 4.6. Morphological and molecular characterization of predominant phosphate solubilizing bacteria

IPB and OPB strains were picked up based on unique colony morphology. These isolates were identified as phosphate solubilizing bacteria and certain strains were further characterized by a series of biochemical reactions following the Bergey’s Manual of Systemic Bateriology [37]. After dilution with sterilized water, transmission electron microscopy (TEM, JEOL JEM-1230, Japan) was employed to characterize the morphology of predominant ones. DNA extraction from bacteria was performed using an EZ Spin Column Bacteria Genomic DNA Isolation 5 kit (SK1201, Sangon, China). Primers 27F (5’-AGAGTTTGATCCTGGCTCAG-3’) and 1492R (5’-GGTTACCTTGTTACGACTT -3’) were used in this study for amplification. The reaction mixture and the PCR cycling conditions followed were by Liu *et al.* [38]. The resulting products were analyzed by electrophoresis in 1% agarose gel, and sequences were determined at Invitrogen Corporation (Shanghai, P.R. China). Sequences were determined by comparison with other sequences in the NCBI databases by blast software. A phylogenetic tree was generated using Paup v.4.0b.8.a Software (the distance matrix was calculated by Kimuras two-parameter model and Bootstrap test sampling 1000 times). The determined 16S rDNA sequences have been deposited in Genebank under accession numbers GQ465222, GQ465223, GQ465224, GQ465225, GQ465226, GQ465227, GQ465228, GQ465229, GQ465230, GQ465231, GQ465232, and GQ465233.

### 4.7. Statistical analysis

All data were analyzed using a SPSS 16.0, Microsoft Excel 2003 and an Origin 8.0. A probability level of 0.05 was considered to be statistically significant.

## 5. Conclusions

Solubilizition of detrital P and mineralization of organic P were two major P bio-relaeasing methods in the West Lake and the Xixi wetlands. High phosphate solubilizing bacteria contents were detected in sediment and the amounts of them were not very relevant to P concentrations. Twelve phosphate solubilizing bacteria, including eight OPBs and four IPBs, which belonged to three different families, were isolated and identified. *Cupriavidus basilensis*, which was reported to have the capability of copper chelation, was found for the first time to have the ability to mineralize OP. Moreover, OPBs grew slower and liberated less orthophosphate than IPBs. The most efficient IPB strain could accumulate over 170 mg L^-1^ orthophosphate, while OPB strain only assembled less than 4 mg L^-1^ orthophosphate in liquid culture, and the biomass of OPB in lecithin media was also lower than that of IPB.

From our findings and conclusions, we recommend that future work should concentrate on investigating the basic generality of phosphate solubilizing bacteria, especially on the genetic and molecular level, in order to find the specific genes acting on P transformation. Different species of phosphate solubilizing bacteria should be identified inasmuch as possible, and relationships between specific bacteria and environmental conditions/eutrophic levels are expected to be discovered. Furthermore, the contributions of phosphate solubilizing bacteria to P transfer can also be analyzed by adding the isolated bacteria into the sediment with sterilization. It is hoped that these potential studies will enhance our awareness of the roles of microorganisms in aquatic systems and the mechanisms of eutrophication.

## Figures and Tables

**Table 1:** Identification of predominant OPB and IPB strains by alignment with 16S rDNA sequences of organisms in the NCBI database.

Strain	Number of nucleotides compared	Closest match^a^ and its accession code	Identity	Accession number
OPB32	730	*Citrobacter freundii* (AB244300)	99%	GQ465231
OPB48	740	*Pseudomonas mosselii* (EU301780)	100%	GQ465232
OPB49	1210	*Cupriavidus basilensis* (AM048887)	96%	GQ465222
OPB51	720	*Cupriavidus basilensis* (AY860244)	98%	GQ465223
OPB57	780	*Citrobacter freundii* (AB184249)	100%	GQ465233
OPB59	700	*Citrobacter freundii* (FJ494899)	100%	GQ465224
OPB72	1060	*Acinetobacter sp.* (EF103565)	99%	GQ465225
OPB98	770	*Burkholderia pyrrocinia* (U96930) *Burkholderia cepacia* (EF095217) *Burkholderia ambifaria* (CP000440)	99%	GQ465226
IPB1	1150	*Enterobacter sp.* (GQ280118)	99%	GQ465227
IPB12	780	*Enterobacter sp.* (EU693568) *Pantoea agglomerans* (EF489443)	99%	GQ465228
IPB15	680	*Enterobacter sp.* (EU693568) *Pantoea agglomerans* (EF489443)	99%	GQ465229
IPB54	680	*Pantoea agglomerans* (EU721614)	100%	GQ465230

^a^ OPB98, IPB12 and IPB54 have two or more closest match, which share the same identity.

**Table 2:** Biochemical characteristics of OPB 49 and OPB 51.

	*Cupriavidus basilensis*	*OPB49*	*OPB51*
Gram	－	－	－
Flagella	＋	＋	＋
Catalase	＋	＋	＋
Oxidase	+	+	+
Nitrate Reduction	/	+	－
Indole test	－	－	－
Adipate	＋	＋	＋
Caprate	＋	＋	＋
Citrate	+	+	+
Glucose	－	－	－
Malate	＋	＋	＋
Maltose	－	－	－
Mannitol	－	－	－
Mannose	－	－	－
Phenylacetate	＋	＋	＋
Mobility	＋	＋	＋

Note: “+” denotes positive, “-” denotes negative, “/” denotes this index does not test or exist
